# Reliability and validity of the modified shuttle test-paeds to measure cardiorespiratory fitness in children

**DOI:** 10.1186/s12887-024-04812-0

**Published:** 2024-05-17

**Authors:** WFM Aertssen, A van de Kamp, LD Jelsma, BCM Smits-Engelsman

**Affiliations:** 1 University for Professionals, Breda, The Netherlands; 2 Clinical and Developmental Neuropsychology, University of Groningen, Groningen, The Netherlands; 3 Department of Health & Rehabilitation Sciences, Faculty of Health Sciences, University, Cape Town, South Africa; 4 Physical Activity, Sport and Recreation, Faculty Health Sciences, North-West University, Potchefstroom, South Africa

**Keywords:** Modified shuttle test-paeds, Cardiorespiratory fitness, Psychometric properties

## Abstract

**Background:**

The Modified Shuttle Test-Paeds (Paeds), a recently developed 10-meter Shuttle run test for aerobic capacity in children. This study aims to investigate the construct validity (known-group and convergent validity) and test-retest reliability of the recently developed test for cardiorespiratory fitness, the Modified Shuttle Test-Paeds (Paeds).

**Methods:**

A total of 144 participants (6–12 y) were tested on the Paeds test, and 84 children were tested on the 20-meter Shuttle Run test (20 m-SRT) to assess construct validity. To evaluate test-retest reliability, 46 children were tested twice on the Paeds.

**Results:**

No sex differences were found, but there was an age effect. A strong correlation was found between Paeds and the 20 m-SRT (r_s_=0.78, *p* < 0.001). The test-retest reliability was good (ICC 0.84; 95% CI 0.74–0.91).

**Conclusion:**

Paeds appears to be a reliable and valid tool for estimating cardiorespiratory fitness in typically developing children aged 6–12 years and has the advantages of being shorter, needing less space, not requiring pacing and being self-motivational. More studies are needed to assess whether children reach an aerobic steady state in three minutes and how much of the results of the Paeds test can be explained by the agility component of the task (turning and grasping or aiming a bean bag). For clinical use, psychometric properties should be studied in various patient groups (e.g., ADHD, DCD, and children with intellectual disabilities).

## Introduction

Physical inactivity, high screen time, sedentary lifestyles, and short sleep durations are current problems in youth and are therefore targets of public health initiatives for children [1]. Physical inactivity leads to lower levels of physical fitness (e.g., aerobic capacity) and subsequently may lead to secondary health problems, such as obesity and cardiovascular disease [2–4]. Good physical fitness is important not only for physical but also for mental health and social-emotional well-being [5–7].

The gold standard for measuring aerobic fitness is VO_2max_. Aerobic fitness is defined as the maximal volume of oxygen (VO_2max_) that can be attained per unit of time and body weight, measured in an exercise involving large muscle groups in laboratory settings by using gas analysis [8]. However, laboratory tests are expensive and mostly not available in clinical practice, schools, or sports environments. When attaining an individual’s maximum oxygen uptake during a laboratory-based test is not feasible, field-based tests can be an alternative for estimating cardiorespiratory fitness. The 20-meter Shuttle Run test (20 m-SRT) is the most frequently used field-based test and can be considered an international health surveillance measure for the pediatric population [9, 10]. However, in a pediatric population, some extra precautions for obtaining valid results should be considered. For a good score on the 20 m-SRT, a running speed at a prescribed speed (pacing) is an essential part of the test performance. Children must run 20 m between 2 beeps. The time between the 2 beeps decreases further in the test. Hence, children must estimate the available time and adapt their running speed accordingly. For some children, this is hard. This pacing problem has been noted by several authors and may be enhanced in clinical groups. In children with Down syndrome, the 20 m-SRT was less effective at predicting VO_2max_ [11]. Cairney et al. (2006) suggested that children with developmental coordination disorders (DCD) stop early because they believe that they are not as good as their typically developing peers [12]. Therefore, psychological, cognitive, and motivational factors could influence outcomes [12, 13]. Hence, there is a need for another valid and reliable field-based test for school-aged children that can be used to estimate maximal oxygen uptake indirectly and that avoids pacing.

The Modified Shuttle Test-Paeds (Paeds), a recently developed 10-meter shuttle run test for aerobic capacity [14, 15], may be a suitable candidate. In the Paeds, children must run for 3 min over a 10-meter distance; they pick up a beanbag on one end, run back and put the beanbag on a tray on the other end, and then run back to pick up another beanbag. The advantages of the test are that synchronizing to an acoustic signal is not needed, it takes less time compared to the 20 m-SRT, it requires less space, and it is a motivational activity for children to gather as many bean bags as possible. A previous study revealed a strong predictive relationship between Paeds and the laboratory-measured peak VO_2peak_, the highest value of which represents the tolerance limit [14].

In addition to the association with the measured VO_2peak_ information, no other psychometric properties of the Paeds have been studied. Milne et al. (2018) recommended that more research regarding psychometric properties is needed before Paeds can be used in clinical practice and in school- and sports environments [14]. In this study, we investigated construct validity (known-group and convergent validity) and test-retest reliability in a group of typically developing children aged 6–12 years. For known-group validity, we examined the relationships between the Paeds and anthropometric characteristics of the children (age and sex). Older children and boys are expected to outperform younger children and girls, respectively [16, 17]. For convergent validity, the association between the Paeds and the.

20 m-SRT was investigated. Based on the similarity of the construct intended to be measured by both tools, a high correlation (> 0.7) was expected. Finally, test-retest reliability was measured, and the smallest detectable change was determined.

## Method

### Design

A cross-sectional design was used to investigate the psychometric properties of the Paeds. Informed consent was signed by the parents. The research was conducted according to the Declaration of Helsinki. Ethical approval was obtained via the ethical committee of the University of Groningen PSY-1920-S-0107.

### Participants

A convenience sample of children aged between 6 and 12 years from primary schools in the Netherlands participated in this study. Children were included in the study if they had no signs of underlying pathologies impeding participation in physical activity, such as cardiovascular (e.g., heart condition), musculoskeletal (e.g., joint or bone problems), metabolic (e.g., diabetes) or neurological (e.g., epilepsy) disorders. To check for eligibility, the parent(s) completed the child’s physical activity readiness questionnaire (PARQ) [18]. Given the expected correlation of at least 0.60, the power analysis for correlation statistics revealed that with a sample size of 30 participants, a statistical power of 95% can be achieved.

### Measures

#### Anthropometric

Weight was measured without shoes using a calibrated electronic scale to the nearest 0.1 kg. Height was measured with a wall-mounted height rod to the nearest 0.1 cm. Body mass index (BMI) was calculated using the formula BMI = weight (kg)/height^2^ (m^2^).

#### The 20-meter shuttle-run test (20 m-SRT) [9]

The 20 m-SRT estimates cardiorespiratory fitness. Participants must run up and down over a distance of 20 m marked with two lines. Children were verbally encouraged during the test to ensure their maximum performance. The intensity of the 20 m-SRT progressively increases, with the pace of the test indicated by audible tones, commencing at a speed of 8.0 km/h, which is increased by 0.5 km/h each stage after the first minute. The child must keep pace with a prerecorded sound signal. The child starts to run at the beep and must be on the other side before the next beep. If the child was unable to keep pace with the sound signal on two consecutive occasions, the test was ended. The number of shuttles (laps) completed was recorded and used in the analysis, and the speed reached in the last stage was used to estimate VO_2max_. Leger’s equation was used to predict VO_2max_ from the 20 m-SRT [9], calculated as follows:

VO_2max_ (mL/kg/min) = 31.025+ (3.238*speed) – (3.248 *age) + (0.1536 *age*speed). The speed (km h-1) corresponds to the stage (8.5 + 0.5 stage number).

#### Modified shuttle test-paeds (Paeds) [14]

For this test, two lines are needed 10 m apart. Behind each line, there is a tray on the floor. Children start behind the line and run after the start signal to the other line to a tray filled with bean bags. The child picks up a beanbag, runs back and places the beanbag in an empty tray, turns, and repeats the task as often as possible in three minutes. The number of transported beanbags was scored as a point. When the child has a beanbag in his/her hand, it counts as half a point. The number of points was used for the analysis.

### Procedure

**Fig. 1:**
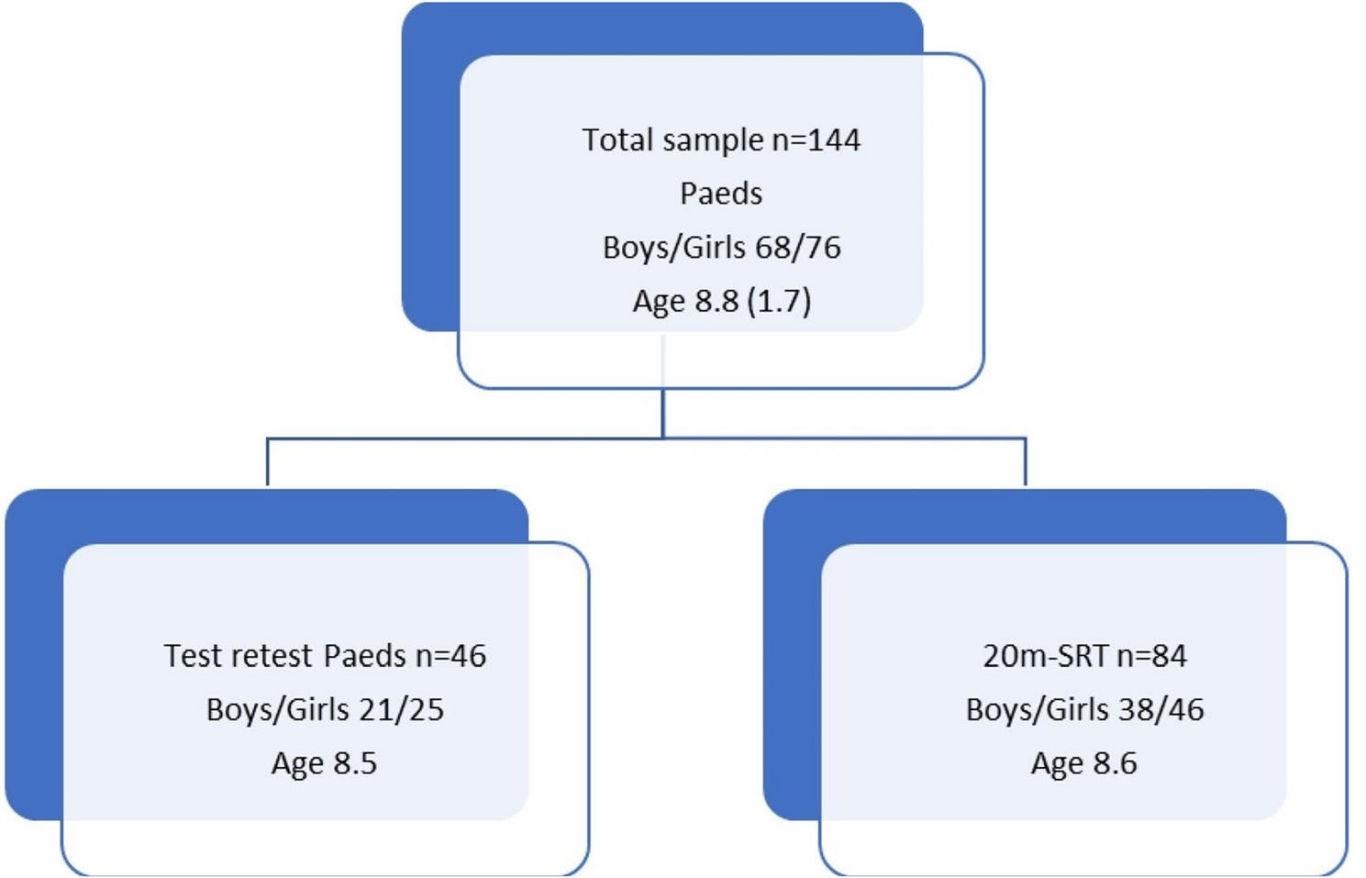
Flowchart of the population within the different research questions

The testing took place at the gym of the participating schools. Children were tested in small groups by trained testers (pediatric physical therapists), and tests took place over two to three days at a similar time of day (see Fig. 1 flowchart). On the first day, height and weight were measured, and the Paeds were administered. On the second test day, some of the children were retested on the Paeds test. On the third test day, some of the children were tested on the 20 m-SRT within three weeks.

### Statistical analyses

The Shapiro–Wilk test showed that the Paeds data and 20 m-SRT VO_2max_ data were normally distributed, but the 20 m-SRT outcomes were not.

For known-group validity, independent t-tests were used to test for possible sex differences, and ANOVA was used to examine age-group differences. To increase the size and balance of the comparative groups, age combinations of 6–7 years and 10–12 years were created.

For convergent validity, Spearman correlation was used to calculate the correlation between the Paeds and the number of laps of the 20 m-SRT. Values < 0.40 are considered low, 0.4–0.7 moderate, and > 0.7 high [19].

For the analysis of test-retest reliability, the intraclass correlation coefficient with absolute agreement (ICC model 2.1 A) and the 95% confidence interval (CI) were determined between the two assessments of the Paeds. An ICC above 0.9 was considered excellent, 0.75–0.9 was considered good, 0.5–0.75 was considered moderate, and less than 0.5 was considered low [20]. The standard error of measurement (SEM) was calculated by dividing the SD_difference_ by the square root of two (SD_difference_/√2) [21]. The SEM provides information about the systematic measurement error. The smallest detectable change (SDC) was determined by multiplying the SD of the difference (SD_difference_ by 1.96) [21]. Bland–Altman plots were constructed to visualize the measurement bias and the limits of agreement (LoA). All the statistical analyses were performed with SPSS version 28. Alpha was set at 0.05.

## Results

### Participant characteristics

In total, data from 144 children (68 boys, 47.2%) were available on the Paeds, and data from 84 children were available on the 20 m-SRT. BMI classification revealed that 84.7% of the children were normal weight, 1 child was obese, 4.9% were underweight, and 9.7% were overweight. The anthropometric data are given in Table 1.

**Table 1:** Participants’ demographics for the total sample and the subsamples

	Total population		Test-retest reliability		Convergent validity	
	*N* = 144		*N* = 46		*N* = 84	
	Mean (SD)	Min–max	Mean (SD)	Min–max	Mean (SD)	Min–max
Age (years)	8.86 (1.58)	6–12	8.46 (1.11)	7–12	8.64 (1.45)	6–12
Height (cm)	140.43 (10.04)	118–167	137.98 (7.73)	126–159	139.15 (11.50)	126–167
Weight (kg)	33.30 (8.48)	20-52.8	31.41 (6.74)	21.4-5.,6	32.50 (8.36)	21.4–52.8
BMI	16.81 (2.21)	13.3–25.7	16.37 (2.35)	13.3–25.6	16.50 (2.10)	13.3–25.7

SD = standard deviation; Min = minimum; Max = Maximum; cm = centimeters; kg = kilogram

### Known-group validity

The test results for age and sex differences are shown in Table 2.

**Table 2:** Cardiorespiratory fitness characteristics per age group, sex and total group

PAEDS	Age groups*		*N*	Mean	SD
	6–7		29	18.6	2.6
*Paeds First Trial*	8		29	19.4	1.7
	9		43	20.0	1.7
	10–12		43	20.3	2.3
	***Gender***		***N***	***Mean***	***SD***
	male		68	19.87	2.23
	female		76	19.56	1.99
	total		144	19.70	2.11
***20 m-SRT***	Age groups**		***N***	***Median***	***Range***
	6–7		17	24	10–52
	8		23	38	20–56
	9		27	34	13–56
	10–12		17	43	13–78
*Laps*	***Gender***				
	male		38	38	15–76
	female		46	30	10–78
	total		84	32.5	10–78
*VO2max*	***Age groups***		***N***	***Mean***	***SD***
	6–7		17	*48.44*	*2.55*
	8		23	*48.15*	*3.04*
	9		27	*46.29*	*3.05*
	10–12		17	*45.57*	*5.40*
	Gender***		***N***	***Mean***	***SD***
	male		38	48.32	3.66
	female		46	46.07	3.56
	total	84		47.09	3.75

* Difference between age groups for the Paeds F(3,140) 5.03, *p* = 0.002

**Difference between age groups for the 20 SRT H(3,80) 9.92, *p* = 0.019

*** Sex difference VO2max: t (1,82) 2.85; *p* = 0.003

**Fig. 2:**
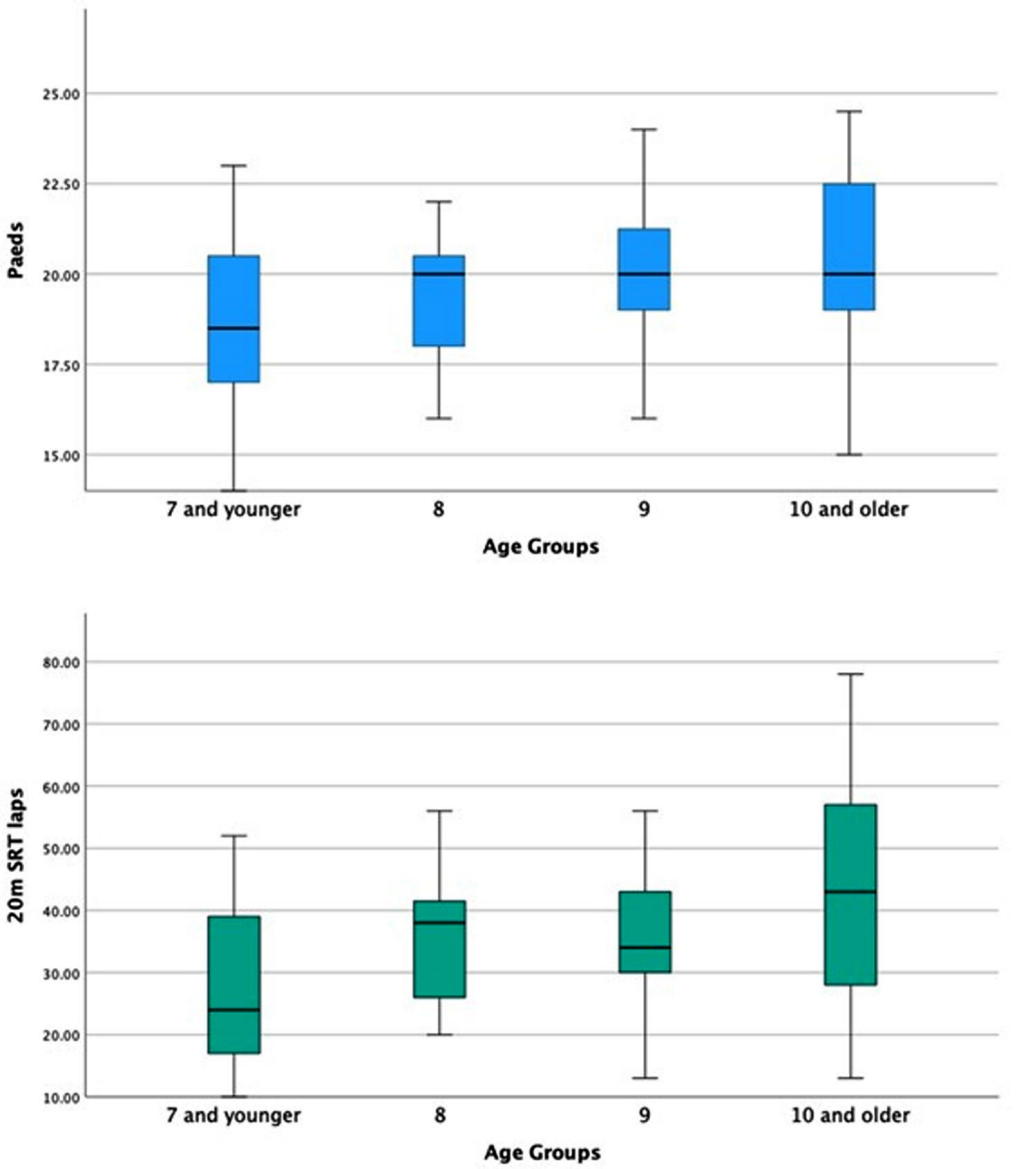
Age differences on the Paeds and the 20 m-SRT

Paeds (F(3,14) = 5.03, *p* = 0.002) and 20 m SRT (H(3,80) 9.92; *p* = 0.019) outcomes differed between age groups. However, the differences between consecutive age groups were small, and for the Paeds, the differences were only significant between the younger group (6–7 years group and 9 years group, *p* = 0.027) and between the younger group (6–7) and the 10 years old, *p* = 0.007). For the 20 m SRT, the differences between the youngest and oldest groups were significant (6–7 years group and 10–12 years group, *p* = 0.012) (Fig. 2).

No sex differences were found for the Paeds (t(1,142)0.88, *p* = 0.38) or 20 m SRT laps (H(1,83)700.5, *p* = 0.12), while for the VO_2peak_, based on the 20 m-SRT, the boys had higher values (t(1,82)2.85, *p* = 0.003) than the girls.

The available sample (*n* = 84) of the 20 m-SRT data showed a VO_2max_ of 46.07 ± 3.56 mL/kg/min for girls and 48.32 ± 3.66 mL/kg/min for boys, which is considered to be within the 60th percentile range [22]. If we use a cutoff for reduced estimated cardiorespiratory function (CRF) of the 20th percentile, 12 children (14.3%) would be classified as having reduced CRF on the 20mSRT (range 1.9–2.1 mL/kg/min below the cutoff value).

### Convergent validity

**Fig. 3:**
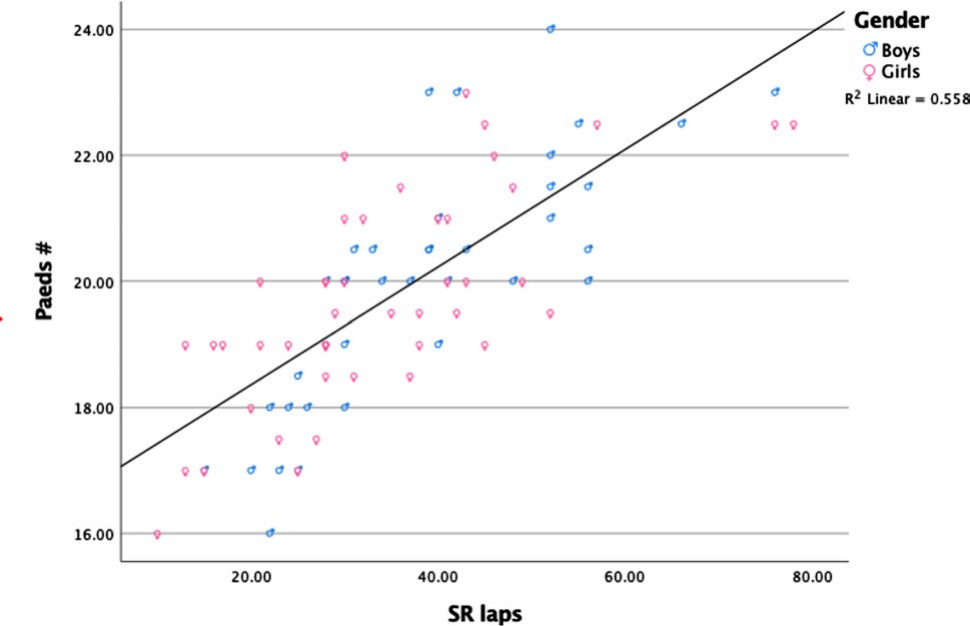
Correlation between the Paeds (# =number of bags) and the 20 m-SRT (laps)

Eighty-four children participated in this part of the study. There was a high correlation between the number of points on the Paeds and the number of laps in the 20 m-SRT (r_s_=0.78, *p* < 0.001). See Fig. 3.

### Test-retest reliability

**Fig. 4:**
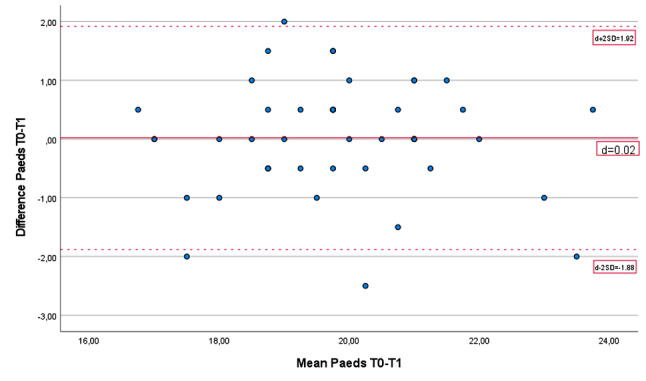
Bland‒Altman plot of the test-retest reliability of the Paeds

Forty-six children participated in the test-retest reliability part of the study. The mean Paeds score of the first test for these children was 19.84 (± 1.67), and that of the retest was 19.82 (± 1.70) points. The mean difference between tests 1 and 2 was 0.02 points. The ICC was 0.84 (95% CI 0.74–0.91), which is considered good. The standard error of measurement (SEM) was 0.67 points. The smallest detectable change (SDC) was 1.86 points. Bland–Altman plots (see Fig. 4) showed a small measurement bias. Most of the results (91.3%) were within the limits of agreement (LoA; 1.92 and − 1.88).

## Discussion

This study extended the knowledge of the psychometric properties of a newly developed tool to measure cardiorespiratory fitness, the Modified Shuttle Test-Paeds (Paeds). For construct validity, we examined known-group validity by examining the association of the Paeds with anthropometric characteristics of study participants and convergent validity by examining the association between the Paeds and the 20 m-SRT. Additionally, the test-retest reliability was investigated.

### Known-group validity

#### Age and sex

The associations between anthropometric characteristics and the Paeds score were significant for age group but not for sex. This effect of age groups showed that overall, the number of points the children obtained was greater when the children were older. However, the changes in scores were small and only significantly different between the youngest age group and the rest of the sample (see Table 2). The results for the 20 m-SRT pointed in a similar direction. This age effect has been extensively documented [16], thus confirming known-group validity.

We also hypothesized that boys would outperform girls, which has been reported on the 20 m-SRT in the literature [17] and corroborates findings on the 20 m-SRT in this study. Boys tend to have more muscle mass than girls during childhood, although it takes until puberty for relative muscle mass to truly increase during the growth spurt [16]. By the middle teens, boys have greater blood hemoglobin concentrations than girls, which will also augment the sex differences in muscle mass and stroke volume in the attainment of higher VO_2max_. However, most of the children in our group were younger, and we did not have Tanner stages to check their pubertal stage. The fact that we only found differences in the 20 m-SRT and not in the Paeds could be because girls can compensate with better agility (turning and grasping or aiming a bean bag) for their lower sprint speed.

### Convergent validity

The convergent validity of the Paeds was examined by comparing the results with those of the 20 m-SRT. The association between the two tests was within the expected range (r_s_=0.78), indicating good convergent validity. Milne et al. (2018) reported a correlation between the measured VO_2peak_ in the laboratory and the Paeds of 0.87 [14]. Although that study had a small sample (*n* = 24) of older children (mean age 12.6), mainly boys (19/5), the values seemed to be in a comparable range.

In the current study, we used two tests intended to estimate cardiorespiratory fitness. Cardiorespiratory fitness is defined as the maximal capacity of the pulmonary and cardiovascular system to take up and transport oxygen to the exercising muscles and of the exercising muscles to extract and use oxygen from the blood for aerobic energy production during *progressive exercise* with large muscle groups up to *maximal exertion* [8, 23]. Thus, cardiorespiratory fitness reflects the overall capacity of physiological systems (cardiovascular, respiratory, metabolic, and neuromuscular) to perform continuous, large muscle group physical activity of *moderate to high intensity for long periods* [10]. Given these definitions (*progressive exercise, maximal exertion*, *high intensity for long periods*), some task constraints of the two tests in relation to the pediatric population need to be considered in more detail. Running in the Paeds takes place at *moderate to high intensity* for 3 min. Whether 3 min is long enough to reach an aerobic steady state in children needs to be studied. It also needs to be examined whether the Paeds test is suitable for children with high levels of fitness. Interestingly, half of the children were also ready with the 20 m-SRT within 3 min. In both tests, we noticed that many children start too fast and may tap into their anaerobic system very, exerting quickly. Moreover, it has been reported that children often do not reach a VO_2_ plateau due to a lack of motivation or lower tolerance for discomfort [24]. However, other studies with large samples of both children and adolescents have shown that those who plateau do not have higher VO_2max_, heart rate, or postexercise blood lactate values than those who do not exhibit a VO_2max_ plateau [25].

During the 20 m-SRT, a child can stop running when (s)he feels discomfort or will be forced to stop if (s)he does not make it to the line in time, which is used to operationalize exertion. In the Paeds, children will not be excluded but tend to slow down if they become tired, so there is no clear point of exertion. Additionally, the Paeds is not a progressive exercise but rather self-paced based on what the child feels (s)he is maximally capable of and how motivated the child is to collect as many bags as possible. Importantly, the behavior tested (running with different task demands) requires not only cardiovascular fitness but also agility to bend, pick a bean bag, and turn as quickly as possible. As mentioned in our introduction, the notion of pacing can be one of the problems when administering 20 m-SRT, which is circumvented in the Paeds. Planning is an aspect of executive functioning [26]. In typically developing children and adolescents, these aspects may not be limiting factors, but in young children and children with motor difficulties such as DCD or children with intellectual disabilities, this may impact the validity of the aerobic tests. Another advantage of Paeds is that it does not have a preprescribed intensity, which makes it more suitable for deconditioned populations. However, in less skilled children, there is a range of other factors in the Paeds that can affect performance that are important to consider. These include running efficiency and turning technique, motivation for continuous activity, and social dynamics if performed in small groups. Future studies will have to evaluate the magnitude of the influence of these factors.

Since the results of a psychometric study may not apply to other patient groups and settings, it is important to investigate the psychometric properties in different age groups, children with different BMIs, and pediatric patient groups. As poor health and reduced motor skills often cooccur, the relationship between Paeds outcomes and motor impairments needs further study. Associating Paeds outcomes with agility measures might shed light on the explanatory power of coordination on the results. In contrast to the 20 m-SRT, in the Paeds, fast turns are important for good performance. The child must start, run, and decelerate before reaching the tray, bend to pick up the beanbag from the tray, turn around, accelerate to run back, etc. It could be that for children with difficulties in agility, such as overweight children or children with neurodevelopmental coordination disorders, this is extra challenging. Specifically, the cutting movement in the turn (deceleration/acceleration) combined with the simultaneous manual task may add another aspect to the task, which is not related to cardiorespiratory fitness.

### Test-retest reliability

Test-retest results confirmed good reproducibility (ICC = 0.84) in school-aged children (6–12 y). Leger et al. (1988) reported a test-retest reliability of *r* = 0.89 for the 20 m-SRT for a young group of children [9], which is comparable to the values of the Paeds in the present study.

The standard error of measurement of the Paeds was 0.67 points, which is small. The smallest detectable change was less than 2 points, which means that the child must transport 2 more beanbags to the tray to indicate improvement (10% of the mean). With a good ICC and small measurement error, the Paeds seems to be a reliable measure for estimating aerobic capacity in young children. Whether this measurement error is small enough for the Paeds to be used for intervention studies needs further study. Most children experienced Paeds as a “game”, which may make the test less discouraging for repetitive use during pre- and postintervention.

### Future research

Although the Paeds is a child-friendly, valid and reliable tool where executive functioning, such as planning of speed, is less needed, the impact of task-specific constraints still needs further study. What is the impact of agility on outcomes given the numerous accelerations, decelerations and turns? Do children reach maximum performance and their VO_2_ plateau given the 3-minute test duration? Is the Paeds sensitive enough to performance change? What are the psychometrics of the Paeds in clinical groups? Additional studies aimed at clinical groups may help to gain more insight into these validity aspects of the Paeds. Comparing the Paeds with other aerobic tests will also provide more information about the validity of the Paeds. Recording heart rate may provide an indication of how close children are to their estimated maximum heart rate, and perceived exertion will provide insight into the perceived level of fatigue. Because the Paeds is a continuous task without elimination, children may become tired and slowdown in the later runs of the tasks. This would mean an opposite pattern of exertion to the 20 m-SRT, since to succeed at higher levels, they need to run laps at a higher speed. In future research, we could investigate this opposite pattern by recording run time or the number of runs per minute. Furthermore, it is important to investigate the feasibility and psychometric properties of the Paeds in clinical groups known with cognitive, motivational and behavior problems, such as children with Down Syndrome and children with ADHD.

There are several limitations to this study. The sample was recruited from available schools close to the researchers. This resulted in a relatively fit sample (Vo2max) with less than 10% of the children classified as overweight or obese. This percentage is lower than expected; the current percentages of overweight and obese children are reported to be approximately 15.5% and 3.6%, respectively, while they were only 6.6% and 1.3%, respectively, in our sample [27]. The level of motor performance, specifically agility, may influence the results, which should be verified in future studies. To gain more information about fatigue and its relationship with aerobic capacity, the ratings of perceived exertion and heartbeats would have provided valuable information.

## Conclusion

Paeds seems to be a reliable and valid estimate of cardiorespiratory fitness in typically developing children aged 6–12 years. The Paeds is highly correlated with the 20 m-SRT, and has the advantages of being shorter, needing less space, not requiring pacing, and being self-motivational. Although the 20 m-SRT is the preferred field test for assessing cardiorespiratory fitness, the Paeds can be used as a valid and playful alternative in pediatric physical therapy settings. In future studies, we advise testing the relationships between the Paeds and the levels of physical activity, agility, heart rate, and perceived exertion. Moreover, the psychometric properties should be investigated in clinical groups, and measurement errors should be investigated for different age groups.

## Data Availability

Data sets generated during the current study are available from the corresponding author on reasonable request. The natural gas production data are available from Drilling Info but restrictions apply to the availability of these data, which were used under license for the current study, and so are not publicly available.
